# Long-Term Effect of a School-Based Physical Activity Program (KISS) on Fitness and Adiposity in Children: A Cluster-Randomized Controlled Trial

**DOI:** 10.1371/journal.pone.0087929

**Published:** 2014-02-03

**Authors:** Ursina Meyer, Christian Schindler, Lukas Zahner, Dominique Ernst, Helge Hebestreit, Willem van Mechelen, Hans-Peter Brunner-La Rocca, Nicole Probst-Hensch, Jardena J. Puder, Susi Kriemler

**Affiliations:** 1 Swiss Tropical and Public Health Institute, Basel, Switzerland; 2 University of Basel, Basel, Switzerland; 3 Department of Human Movement Science, Maastricht University, Maastricht, The Netherlands; 4 Institute of Exercise and Health Sciences, University of Basel, Basel, Switzerland; 5 University Children's Hospital Würzburg, Wuerzburg, Germany; 6 Department of Public and Occupational Health and EMGO+ Institute, VU University Medical Center Amsterdam, Amsterdam, The Netherlands; 7 Department of Cardiology, Maastricht University Medical Center, Maastricht, The Netherlands; 7 Service of Endocrinology, Diabetes and Metabolism, Centre Hospitalier Universitaire Vaudoise, University of Lausanne, Lausanne, Switzerland; 9 Institute of Social and Preventive Medicine, Department Physical Activity and Health, University of Zürich, Zürich, Switzerland; NIDDK/NIH, United States of America

## Abstract

**Background:**

School-based intervention studies promoting a healthy lifestyle have shown favorable immediate health effects. However, there is a striking paucity on long-term follow-ups. The aim of this study was therefore to assess the 3 yr-follow-up of a cluster-randomized controlled school-based physical activity program over nine month with beneficial immediate effects on body fat, aerobic fitness and physical activity.

**Methods and Findings:**

Initially, 28 classes from 15 elementary schools in Switzerland were grouped into an intervention (16 classes from 9 schools, n = 297 children) and a control arm (12 classes from 6 schools, n = 205 children) after stratification for grade (1st and 5th graders). Three years after the end of the multi-component physical activity program of nine months including daily physical education (i.e. two additional lessons per week on top of three regular lessons), short physical activity breaks during academic lessons, and daily physical activity homework, 289 (58%) participated in the follow-up. Primary outcome measures included body fat (sum of four skinfolds), aerobic fitness (shuttle run test), physical activity (accelerometry), and quality of life (questionnaires). After adjustment for grade, gender, baseline value and clustering within classes, children in the intervention arm compared with controls had a significantly higher average level of aerobic fitness at follow-up (0.373 z-score units [95%-CI: 0.157 to 0.59, p = 0.001] corresponding to a shift from the 50th to the 65th percentile between baseline and follow-up), while the immediate beneficial effects on the other primary outcomes were not sustained.

**Conclusions:**

Apart from aerobic fitness, beneficial effects seen after one year were not maintained when the intervention was stopped. A continuous intervention seems necessary to maintain overall beneficial health effects as reached at the end of the intervention.

**Trial Registration:**

ControlledTrials.com ISRCTN15360785

## Introduction

Clinical markers of chronic disease in adulthood like atherosclerosis, obesity and osteoporosis are due to lifelong processes that originate in childhood with physical inactivity and low aerobic fitness as key players in the high burden of chronic disease.[1] In children, both factors are associated with increasing prevalence of cardiovascular risk factors[2], [3] even independent of body weight.[4] There is strong evidence that high aerobic fitness and physical activity protect adults - with or without increased body fat - from increased morbidity and mortality.[5] Yet, aerobic performance levels in youth have almost globally decreased over the last decades[6] and not even half of the children meet physical activity recommendations.[7]

The importance of primary prevention by promoting physical activity in general[8] as well as in the scope childhood obesity has become indisputable,[9] as most pediatric obesity treatment interventions are marked by small changes in adiposity,[10] a substantial relapse rate and by a strong tracking of overweight into adulthood.[11] School-based intervention studies promoting a healthy lifestyle have shown favourable immediate health effects.[12] However, there is a striking paucity of information on long-term follow-up especially of high-quality randomised, controlled, theory driven trials that had shown efficacy at the end of their interventions.[12], [13] Therefore, we report the 3-year follow-up results of a cluster-randomised, controlled trial (“Kinder- und Jugendsportstudie”; KISS) comparing a school-based stringent physical activity program to traditional physical education during one school-year. This trial has shown beneficial short-term effects on aerobic fitness, physical activity, body fat and a composite cardiovascular risk factor score.[14]

## Methods

### Ethics statement

The study was approved by the ethics committees of the University of Basel and the Swiss Federal Institute of Technology Zurich, as well as by the Cantonal Ethical Committee of Aargau, Switzerland. Written informed consent was provided by at least one parent and all children gave their assent for participation to the whole study and specifically for the blood withdrawal. The protocol for this trial and supporting CONSORT checklist are available as supporting information; see Checklist S1 and Protocol S1.

### Design and study population

The design and the short-term effects have been published previously.[14], [15] Briefly, this cluster randomized controlled trial with a multi-component physical activity intervention was performed between August 2005 and July 2006 in two of 26 provinces of Switzerland comprising 10% of the Swiss population. Of the 95 schools fulfilling our stratification criteria, i.e. rural versus urban localisation, and a prevalence of 10–30% children from other ethnicities as in the Swiss population, and, for practical reasons, the presence of at least one first and fifth grade class per school, we randomly selected 15 schools and assigned them in a 3∶2 ratio to an intervention (n = 9 schools) and a control arm (n = 6 schools). Randomization by school was done to avoid a contamination of treatments and was performed based on a computer-generated random-number table which was in the hands of a person not involved in the study. All measurements were performed at school. The younger children, now in fifth grade, were contacted through and tested in their respective schools (June 2009). The former fifth graders now attending different secondary schools/colleges were contacted individually and testing was done in an easily reachable, centrally located school (August to November 2009). For participation in the baseline and post-intervention testing, children received a swim towel with the KISS logo and parents received a written report about individual results of their child. Participants in the follow-up received a small kite (younger children) and a gift voucher (value 30 CHF for younger children or 50 CHF for older children).

### Intervention

The intervention was targeted both at the cluster and the individual level and was based on a socio-ecological conceptual model focusing on increasing daily physical activity as previously described.[15] Briefly, children in both groups had three physical education lessons per week (45 minutes each) given by the usual classroom teachers. The intervention group had two additional physical education lessons (45 minutes each) on the remaining school days that were taught by physical education teachers. The curriculum for all physical education lessons for the intervention group was prepared by a team of expert physical education teachers and the same curriculum aiming at increasing quality of physical education and quantity of at least moderately intense physical activity was provided to all intervention classes. In addition, three to five short activity breaks (two to five minutes each) were introduced every day during academic lessons, comprising motor skill tasks such as jumping or balancing on one leg. The children also received daily physical activity homework of about 10 minutes. Children and parents of the control group were not informed about the existence of the intervention program in other schools. The teachers in the control group knew about the intervention arm, but were not informed about its content. After the intervention, none of the schools continued to provide additional physical education.

### Outcome measures

All measures were taken exactly the same way at baseline, post-intervention and at follow-up.[14], [15] Blinding of the assessors at follow-up was fulfilled, except for waist circumference and skinfold assessment measured by the same persons as before. As in the initial analyses, primary outcome measures included the sum of four skinfolds, aerobic fitness, physical activity and quality of life. Secondary outcome measures included body mass index (BMI) and a cardiovascular risk score comprising all variables of the metabolic syndrome. Overweight was calculated based on WHO criteria.[16] Skinfold thickness was measured in triplicate to the nearest 0.5 mm with Harpenden calipers (HSK-BI, British Indicators, UK). The sum of four sites (triceps, biceps, subscapular and suprailiacal) was calculated.[17] Aerobic fitness was determined using the 20 m shuttle run test.[18] Physical activity was monitored by an accelerometer (MTI/CSA 7164/GT1M, Actigraph, Shalimar, FL, USA) which was worn continuously around the hip for 7 weekdays during each measure period. The sampling epoch was set at one minute. Time periods with over 15 minutes of continuous zero values were omitted. An individual child's physical activity data were included if at least three weekdays of measurements with a minimum of 12 hours and one weekend day of at least 10 hours were recorded.[19] Physical activity was expressed as average counts/min (cpm) and moderate-vigorous physical activity (MVPA) as minutes above 2000 cpm (which is equivalent to walking at about 4 km/h). A physical activity questionnaire was used to assess children's sports club and leisure time sports participation, as well as parental support for and attitude towards physical activity. Quality of life was assessed by the child health questionnaire[20] distributed at school in coded envelopes and completed by the child, if necessary, with the help of the parents. Blood pressure was measured at the right arm five times after a resting period of five minutes using an automated oscillograph (Oscillomate, CAS Medical Systems, Branford, CT, USA). The mean of the three measurements with the smallest variation was taken and then z-transformed.[21] Blood was drawn in the morning while fasting for measurements of glucose, insulin, and lipids as previously described[15] and a composite cardiovascular risk score[22] was computed by averaging the z-scores of all components of the metabolic syndrome (waist circumference, blood pressure (mean of systolic and diastolic blood pressure z-score), glucose, inverted high density lipoprotein-cholesterol (HDL), and triglycerides). Skewed data were ln-transformed. Z-scores were derived from published age- and gender-specific norm values for BMI[23] and blood pressure.[21] The remaining variables were z-transformed using grade- and gender-specific means and standard deviations derived from the whole sample at each measurement period. Reported time spent in sport clubs (in min/wk), leisure-time physical activity (in min/wk) and parents' support for or attitude towards physical activity in a 5-point Likert scale were assessed by questionnaire.

### Statistical analysis

Children with both, baseline and follow-up data were included in the analysis and defined as “participants”. Baseline comparisons were done using a multilevel linear or logistic regression model with school as random effect using participation (1 = participants vs. 0 = non-participants), group (1 = intervention vs. 0 = control group) and the interaction participation × group as explanatory variables. Analyses of a selection bias for physical activity measures were done using multilevel linear regression models adjusting for sex and grade, comparing children with and without physical activity data for the other primary outcomes. We also used inverse probability weighting based on baseline characteristics (i.e., sex, age, initial fitness, parental education level) and stratifying weight models by group, to assess the direction in which effect estimates might have been biased by differential missing of data (including missing data due to non-participation). For each of the main outcomes, separate weight models were derived. To facilitate interpretation, descriptive results are reported on the original scale according to group and participation status, but all statistical comparisons were done using age and sex-specific z-scores. We used multilevel linear models with z-scores at follow-up as dependent variables, group, gender and grade as fixed factors, the respective baseline z-score as covariate. As BMI and pubertal stage are known to influence aerobic fitness, physical activity and their changes the baseline model was further adjusted by change in BMI z-score and change in Tanner stage. The original school class was the smallest cluster in the sampling design and therefore used as random effect. We did not adjust for multiple comparisons. We did not add a second cluster adjustment for the new class of the older age group since the students were spread into a large number of new classes. We did not include pubertal stage, migrant or socio-economic status, in the final model since their addition to the model did not change the results (data not shown). For each outcome measure, the size of the intervention effect is reported as difference in its average z-scores at follow-up and Cohen effect sizes between the intervention and control group after adjustment for grade, gender, baseline values and clustering within the original school classes. In secondary analyses, potential interactions of the intervention with gender, grade, or baseline BMI, divided into two at the median were assessed and subgroup analyses by gender and grade were performed for the primary outcomes. In an additional step, we also included the group-specific participation propensity scores centered at the respective participation rates to the model in order to adjust for differences in baseline characteristics. According to our original calculation, primarily performed for the outcome of the shuttle run test but equally valid for the other outcomes, a sample size of 360 children at the three year follow-up was sufficient to detect a true mean effect size of half a standard deviation with 79% probability for an intracluster correlation (ICC) within schools of 0.10 and with 90% probability for an ICC of 0.06. A new power calculation, backed up by a Monte Carlo simulation, revealed that our original calculation was too conservative. With 16 intervention and 12 control classes that would each contribute at least 10 children for the 3-year follow-up (n = 280), we would actually already have had a power of 80% to detect a true effect size of 0.5 standard deviations for an ICC of 0.1, and a power of 89% for an ICC of 0.06. The actual ICCs in the follow-up were even lower.

## Results

**Figure 1:**
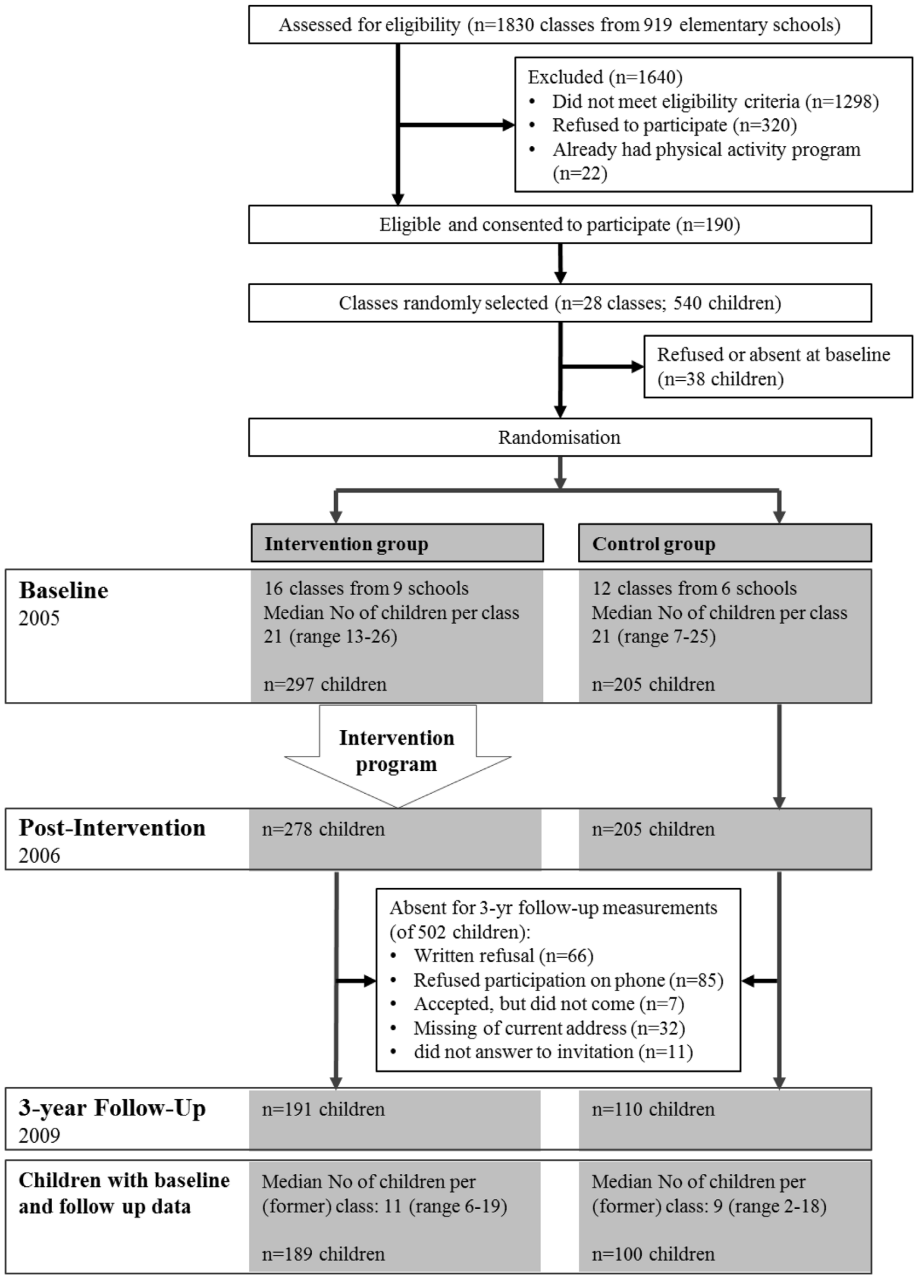
Flow of individual participants through study with outcome measures. Post-intervention results have been published elsewhere [14].

Figure 1 provides sample size information and Table 1 baseline characteristics stratified by allocation arm and participation at follow-up. Characteristics at follow-up are given in Table 2. Overall participation in the 3-year follow-up assessments was 289 (58%) children of the original baseline sample. Most dropouts at follow-up were caused by non-willingness to participate at the measurements. Participation among groups was not different among the younger and older age group, but non-participation was much more common for the older age group irrespective of group (78% of the intervention group and 66% of the control group of non-participants were from the older age category). There were no major differences in baseline characteristics between participants and non-participants at follow-up except for z-scores of sum of four skinfolds, BMI and waist circumference which were lower in participants than in non-participants. The only significant group × participation interaction existed for BMI z-scores, i.e. with lower values for participants of the intervention compared to controls. Missing values mainly occurred in blood and accelerometer parameters, mainly including invalid accelerometer recordings due to too short wearing time. However, there were no statistically significant differences in any of the primary outcomes between children with and without blood samples and between children with and without valid accelerometer data.

**Table 1:** Baseline characteristics of children according to treatment arm and participation at follow-up. Values are means (SD) unless stated otherwise.

		First graders		Fifth graders		Baseline differences between participants and non-participants^a^		
		INT (n = 108)	CON (n = 65)	INT (n = 81)	CON (n = 35)			
		Mean (SD)	Mean (SD)	Mean (SD)	Mean (SD)	p_part_	p_group_	p_group × part_
Age (years)	Participants	6.9 (0.3)	6.9 (0.3)	10.9 (0.5)	11.1 (0.6)	0.07	0.29	0.63
	Non-participants	7 (0.3)	6.9 (0.4)	11.1 (0.6)	11.3 (0.6)			
Height (cm)	Participants	122.5 (5.2)	122.8 (5.6)	144.9 (7.6)	147.2 (7.5)	0.62	0.99	0.34
	Non-participants	124.9 (5.1)	121 (5.2)	145.5 (5.6)	146.7 (7.4)			
Weight (kg)	Participants	24.4 (3.7)	23.6 (4.1)	37.3 (7)	37.4 (7.8)	0.02	0.39	0.33
	Non-participants	25.8 (7.6)	24.8 (4.9)	38.5 (8)	39.9 (8.7)			
Gender, %girls	Participants	51 (47%)	36 (55%)	48 (59%)	23 (66%)	0.04	0.47	0.22
	Non-participants	13 (65%)	19 (79%)	41 (51%)	27 (39%)			
Overweight (%)^b^	Participants	27 (25%)	9 (14%)	15 (19%)	5 (14%)	<0.001	0.1	0.02
	Non-participants	3 (15%)	11 (46%)	20 (25%)	20 (29%)			
Prepubertal/early-pubertal/pubertal^c^	Participants	108/0/0	65/0/0	44/36/1	20/12/3	0.22	0.98	0.68
	Non-participants	24/0/0	36/0/0	36/40/4	33/21/3			
Migrants (%)^d^	Participants	34 (31%)	16 (25%)	16 (20%)	5 (14%)	0.01	0.55	0.13
	Non-participants	10 (50%)	15 (63%)	25 (31%)	20 (29%)			
No formal parental education (%)	Participants	9 (8%)	2 (3%)	2 (2%)	3 (9%)	0.2	0.09	0.31
	Non-participants	3 (15%)	2 (8%)	17 (21%)	8 (12%)			

a Main effects of group and participation and potential interactions between the two age-group and gender specific z-scores were assessed using mixed linear or logistic regression models including indicator variables for participation (part) and group (intervention (INT) vs. controls (CON)), and an interaction term group × participation as well as a random effect for original school class.

b Categorization based on WHO z-scores.

c Pubertal stages are based on Tanner stages: prepubertal (Tanner 1), early pubertal (Tanner 2 and 3), pubertal (Tanner 4 and 5);

d both parents from Eastern or Southern European countries, Africa, Asia, Central or South America, or other less developed countries.

**Table 2:** Follow-up characteristics of participating children according to treatment arm. Values are means (SD) unless stated otherwise.

	First graders		Fifth graders	
	INT (n = 108)	CON (n = 65)	INT (n = 81)	CON (n = 35)
	Mean (SD)	Mean (SD)	Mean (SD)	Mean (SD)
Age (years)	10.6 (0.3)	10.6 (0.4)	15.0 (0.5)	15.1 (0.6)
Height (cm)	145.0 (7.0)	144.1 (6.9)	167.6 (9.8)	167.3 (7.9)
Weight (kg)	37.7 (7.6)	35.8 (8.6)	58.4 (9.7)	56.9 (11.1)
Gender, n (%) girls	51 (47%)	36 (55%)	48 (59%)	23 (66%)
Overweight (%)^a^	32 (31%)	9 (14%)	16 (20%)	7 (20%)
Pubertal stages^b^, n (%)				
Prepubertal	54 (50%)	32 (49%)	1 (1%)	0 (0%)
Early pubertal	51 (47%	32 (49%)	18 (22%)	9 (26%)
Pubertal	3 (3%)	1 (2%)	62 (77%)	26 (74%)
Migrants, n (%)^c^	34 (31%)	16 (25%)	16 (20%)	5 (14%)
No formal parental education, n (%)	9 (8%)	2 (3%)	2 (2%)	3 (9%)

a Categorization based on WHO z-scores.

b Pubertal stages are based on Tanner stages: prepubertal (Tanner 1), early pubertal (Tanner 2 and 3), pubertal (Tanner 4 and 5);

c both parents from Eastern or Southern European countries, Africa, Asia, Central or South America, or other less developed countries.

### Primary and secondary outcomes

**Figure 2:**
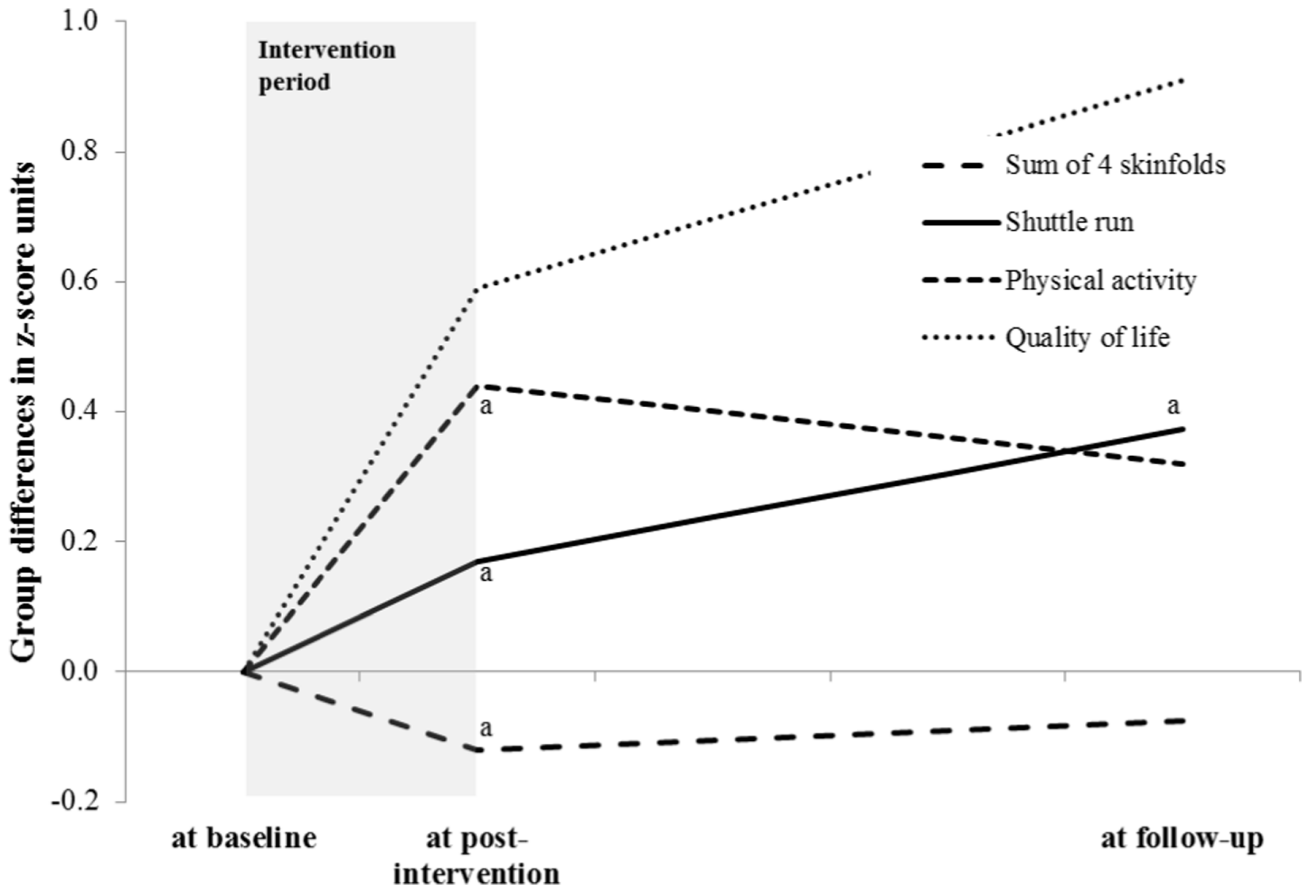
Schematic illustration of the differences for the four primary outcomes between the intervention and the control group at baseline, after nine months physical activity intervention, and three years after cessation of the intervention. Post-intervention results derive from previously published results with different sample sizes [14] (for body fat (n = 485 at post-intervention/n = 293 at follow-up); fitness (n = 472/n = 281); physical activity (n = 303/n = 145); quality of life (n = 427/n = 191)). ^a^significantly different values in favour of the intervention group compared to the control group.

Long-term results of the primary and secondary outcomes are presented in Table 3 and Figure 2. Compared to controls, children in the intervention group showed a significant higher aerobic fitness in the shuttle run by 0.373 z-score units (95% CI 0.157 to 0.590), equivalent to a shift from the 50^th^ to the 65^th^ percentile. This effect was found in 1^st^ and 5^th^ graders of both sexes and corresponded to an average difference of improved running distance of about 150 m at the highest speed reached during the test between the two groups. Children of the intervention group continued to increase their fitness from post-intervention to the follow-up while controls lost performance after the intervention had finished. The results were similar, even after adjustment for changes in BMI z-score and Tanner stage from baseline to follow-up (Table 4). Children from the intervention showed a trend towards higher levels of physical activity. Cohen's d effect sizes were 0.35 for total physical activity and 0.65 for aerobic fitness, respectively. Intervention effects on primary outcomes were comparable in children with and without complete physical activity data (all interaction terms p>0.4). The remaining primary and secondary outcome variables were not significantly different among groups nor among subgroups by gender or grade. This was also true for the variables that showed beneficial effects in favour of the intervention at the end of the intervention period, including sum of four skinfolds, physical activity and the cardiovascular risk score with one exception. The sum of 4 skinfolds and waist circumference at follow up were significantly lower in the intervention compared to the control group in the 5^th^ graders by 12 and 10%, respectively (both p<0.03). The ICCs were ≤0.1 indicating a low level of clustering within school classes. Secondary analyses that involved the study of potential effect modifications by gender, grade, or baseline BMI (being dichotomised at the median) did not show any significant result. Inverse probability weighting to adjust for differential missing of data among groups led to slight increases in beneficial effects while non-significant associations showed changes in both directions. The inclusion of the propensity score led to stronger effects for aerobic fitness (β-coef: 0.64 (95%CI: 0.30 to 0.98); p<0.001) and significant intervention effects for the sum of four skinfolds (−0.42 (−0.68 to −0.15); p = 0.002) and waist circumference (−0.37 (−0.65 to −0.09); p = 0.009). Reported time spent in sports club was significantly higher in the intervention than the control group (reported difference: 72 min per week (95% confidence interval: 10 to 133; p = 0.022)) while leisure-time physical activity, parents' support for, or attitude towards physical activity did not explain group differences (all p>0.05).

**Table 3:** Outcome measures of the three year follow-up in children with and without physical activity intervention aimed at increasing physical activity, fitness and at reducing body fat and a cardiovascular risk score. Values at baseline and follow-up are unadjusted means (SD).

Outcome			Baseline	Follow up	Adjusted group difference at follow-up*				
		n	mean (SD)	mean (SD)	Coefficient (95% CI)	Effect size	P value	ICC	df_err_
Sum of 4 skinfolds (mm) b, d	INT	191	30.9 (11.4)	42.1 (19.3)	−0.076 (−0.222 to 0.069)	−0.23	0.30	<0.01	288
	CON	102	27.8 (10.5)	39.7 (22.3)					
Aerobic fitness (stages) b	INT	181	5.3 (2.3)	6.8 (2.2)	0.373 (0.157 to 0.590)	0.62	0.001	0.02	276
	CON	100	5.4 (1.9)	6.2 (2.1)					
TPA (cpm)	INT	89	729 (174)	544 (208)	0.320 (−0.012 to 0.651)	0.35	0.06	<0.01	140
	CON	56	796 (164)	569 (201)					
MVPA (min/d)	INT	89	89.5 (27.8)	61.5 (28.1)	0.143 (−0.204 to 0.490)	0.16	0.42	0.01	140
	CON	56	98.9 (28)	66.9 (32.5)					
QoL – physical	INT	136	54.8 (5.9)	53.8 (7.3)	0.910 (−1.473 to 3.293)	0.02	0.45	0.03	186
	CON	55	53.4 (7.1)	52.5 (7)					
QoL – psychological	INT	136	53.3 (6.8)	53 (6.8)	1.424 (−0.661 to 3.509)	0.03	0.18	<0.01	186
	CON	55	53.4 (6.3)	51.7 (9.6)					
BMI (kg/m^2^) ^a^	INT	194	16.9 (2.2)	19.1 (2.8)	0.010 (−0.130 to 0.151)	0.03	0.88	<0.01	291
	CON	102	16.1 (2.1)	18.2 (3.3)					
Cardiovascular risk score c	INT	145	−0.016 (0.466)	0.013 (0.512)	−0.003 (−0.208 to 0.201)	−0.02	0.98	0.29	193
	CON	53	0.013 (0.544)	−0.002 (0.545)					
Waist circumference (cm) b	INT	189	56.8 (6.2)	64.7 (7.3)	−0.051 (−0.195 to 0.092)	−0.15	0.48	<0.01	286
	CON	102	55.3 (5.5)	63.3 (7.9)					
Systolic blood pressure (mmHg) c	INT	193	102.9 (8.9)	110.1 (10.8)	0.279 (−0.001 to 0.559)	0.57	0.05	0.17	289
	CON	101	101.2 (7.8)	105.3 (9.6)					
Diastolic blood pressure (mmHg) c	INT	193	61.1 (7.7)	67 (7.9)	0.146 (−0.054 to 0.347)	0.45	0.15	0.11	289
	CON	101	60.4 (7.2)	64.4 (7.9)					
Glucose (mmol/l) b	INT	140	4.5 (0.4)	4.8 (0.4)	−0.004 (−0.401 to 0.394)	0.00	0.99	0.16	182
	CON	47	4.5 (0.5)	4.7 (0.6)					
HDL cholesterol (mmol/l) b, d	INT	145	1.6 (0.3)	1.5 (0.3)	0.147 (−0.154 to 0.449)	0.24	0.34	0.07	193
	CON	53	1.6 (0.3)	1.4 (0.4)					
Triglycerides (mmol/l) b,d d	INT	141	0.6 (0.2)	0.8 (0.4)	0.143 (−0.265 to 0.550)	0.19	0.49	0.19	185
	CON	49	0.6 (0.3)	0.8 (0.4)					

* Differences in average change and 95% confidence intervals (CI) are the differences between intervention (INT) and controls (CON) after adjustment by mixed-model regression analysis for grade, gender, baseline value of the outcome and cluster (class). TPA = total physical activity; cpm = counts per minute; MVPA = moderate and vigorous physical activity, QoL = Quality of Life; BMI = body mass index; HDL = high-density lipoprotein; ICC = intracluster correlation coefficient; df_err_ = error degrees of freedom.

Z-Scores are based on ^a^WHO references,

b internal references,

c CDC references,

d ln-transformed.

**Table 4:** Additional analyses for aerobic fitness when additionally adjusted for change in body mass index or change in pubertal stage. Values at baseline and follow-up are unadjusted means (SD).

Outcome			Baseline	Follow up	Adjusted group difference at follow-up*				
Aerobic fitness (stages)		n	mean (SD)	mean (SD)	Coefficient (95% CI)	Effect size	P value	ICC	df_err_
adjusted for grade, gender, and cluster	INT	181	5.3 (2.3)	6.8 (2.2)	0.373 (0.157 to 0.590)	0.62	0.001	0.02	276
	CON	100	5.4 (1.9)	6.2 (2.1)					
+ change in BMI z-score	INT	175	5.4 (2.3)	6.9 (2.2)	0.279 (0.067 to 0.490)	0.51	0.01	0.02	255
	CON	86	5.6 (1.9)	6.5 (2.1)					
+ change in Tanner stage	INT	177	5.4 (2.3)	6.9 (2.1)	0.343 (0.111 to 0.574)	0.59	0.004	0.04	263
	CON	93	5.5 (1.9)	6.3 (2.1)					

*Differences in average change and 95% confidence intervals (CI) are the differences between intervention (INT) and controls (CON) after adjustment by mixed-model regression analysis for grade, gender, baseline value of the outcome and cluster (class). ICC = intracluster correlation coefficient; df_err_ = error degrees of freedom. Z-scores are based on internal references.

## Discussion

At the current 3-years follow-up of a school-based physical activity intervention over nine months in primary school, aerobic fitness remained significantly higher in favour of the intervention group. The other initially seen beneficial effects on physical activity and a composite cardiovascular risk score were not sustained, except for body fat in the fifth graders that remained lower in the intervention compared to the control group. Reported time spent in sports club during the follow-up period was higher in the intervention group than in controls, while other explanatory factors such as reported leisure-time physical activity and parental attitude towards physical activity were not. This study is one of the few long-term follow-ups of school-based lifestyle interventions to shed light on sustainability of such programs.[12]

The increase in aerobic fitness in favour of the intervention group three years after the end of the intervention, equivalent to a shift from the 50^th^ to the 65^th^ percentile is higher than immediately after the intervention, where it was equivalent to a shift of 5 percentile units. This is due to both a further increase in aerobic fitness in the intervention group and a reduction of aerobic fitness in the control group. Importantly, the differences remained relevant independent of pubertal stage, BMI and their respective changes excluding different developments in body composition or puberty over the study period as confounder. A difference in 150 m of running distance at the highest speed translates into a difference in VO2max of 5 ml/kg/min or 5%. The decrease in the control group corresponds well to the internationally reported decline of youth fitness by 0.43% per year over the last two decades.[14], [24] Such an improved aerobic fitness is clinically meaningful and from a Public health respect most important, if one considers that an increase of aerobic fitness of 5% in adults translates into 6–7% lower risk for cardiovascular events and mortality.[25]

In the participants of our intervention, some behavioural changes – possibly based on positive experiences by the intervention - occurred. Time spent in sports club during the follow-up was higher in the intervention compared to the control group. Even more importantly, these behavioural changes resulted also in some health enhancing effects such as increased aerobic fitness. Part of the maintained effects may also be related to an increased quality and quantity of the physical education at least in the younger age group. This younger age group attended the same school as during the intervention. Of note, 70% of the teachers and 90% of the children wanted daily physical education to continue after the intervention had stopped.[14]

Children's physical activity levels at follow-up were not different among groups, although there was a trend that pointed towards higher levels in children from the intervention compared to controls. This is consistent with a very similar study,[26] but in contrast to other long-term follow-up studies in which reported leisure-time physical activity was still increased three to 20 years after the intervention.[27]–[30] It is possible that the validity of these studies which were based on activity assessments by questionnaires may have been hampered by factors such as social desirability and reporting or selection bias as these few programs did not measure or show persistent effects on aerobic fitness.[31], [32] The lack of significant physical activity results in our study might reflect insufficient power due to a considerable dropout and non-compliance to wear the accelerometer, even in the children that attended the follow-up assessment. Although our observed intervention effect on physical activity lacks statistical significance, its effect size of d = 0.35 would be relevant if it reflected the truth.

Our intervention had initially resulted in an improved body composition with less body fat and lower cardiovascular risk scores that included all components of the metabolic syndrome immediately after the intervention. Unfortunately, this effect was only sustained for the sum of four skinfolds at follow-up for the 5^th^ graders. Only two controlled study in children with a similar follow-up duration and after a one[26] or six[31], [33] year intervention period had reported persistent beneficial effects on blood pressure,[26], [33] blood lipids[31] and glucose metabolism[26] while the few studies that measured long-term effects lost their beneficial effects on adiposity or cardiovascular risk factors.[29], [34], [35] Although one has to be careful with interpreting these data because of methodological limitations and high dropout rates in many of these studies, the maintenance of intervention programs seem necessary over longer time spans, preferably over the whole school period, to have sustained global health effects.

Process evaluation models like the RE-AIM (Reach, Efficacy, Adoption, Implementation and Maintenance of an intervention)[36] that describe the overall health impact of an intervention should always be considered as statistical probabilities or effect sizes on their own do not fully define the real public health impact of an intervention. This important procedure is now taken up by some leading groups in the field,[37], [38] although we were not able to perform it due to financial constraints. In our example with KISS, effect sizes for aerobic fitness and physical activity were relatively large, but its impact is only meaningful if the intervention also reaches a significant part of the population of children and their families. An example in this respect is a small efficacy trial that was performed in a few schools of one province in Canada documenting beneficial effects of a school-based lifestyle intervention.[39] As response to the results the program was then widely implemented throughout the province with the help of the provincial government that mandated 30 minutes of daily physical activity in schools by law. Indeed, the Swiss government has, in part based on results of the KISS study established a National program (Youth and Sport Kids 5–10) that supports schools in establishing additional physical activity programs in primary school by educating classroom teachers to ameliorate quality of physical education teaching and in concert providing financially support. These political steps are promising although their effectiveness has to be proven in the future.

### Limitations and strengths

Similarly to every long-term follow-up of school-based intervention studies published so far, the most important limitation of the current study is the considerable dropout rate. It has to be acknowledged that more obese children and those with a migrant background dropped out although other participants' and non-participants' baseline characteristics did not systematically differ. Nevertheless, we cannot rule out the presence of some selection bias. We tried to account for this possible bias by adding a propensity score to our model (to adjust for differential participation) showing that our results remained the same despite adjustment for participation differences. This is especially true for BMI z-scores, for which we had a participation bias in favour of initially leaner children being more prevalent in the intervention than in the control group. Yet, there were no group differences for participation in the other more precise obesity variables such as waist circumference and the sum of the four skin folds and the intervention effects on aerobic fitness remained significant even after adjusting for BMI z-scores (Table 4), and the results did not change with the inclusion of propensity scores for participation. In terms of level of dropout, our study is comparable to other studies of similar length of follow-up,[26], [35] but persistent participation was still much higher than in long-term follow-ups of extended time windows.[30], [31] We did everything to maximize participation: careful update of addresses, offering of multiple test dates, testing during official school time to motivate children, multiple attempts of motivational inputs given by teachers and the KISS staff, and gift vouchers of choice (books, clothes, sport gear, music, jewellery) were provided. Still, these consistently high dropout rates have to be considered when interpreting the results and in future power analyses especially when adolescents with their “unpredictable attitudes” are involved. This may indeed be one or even the major reason explaining the lack of long-term follow-up studies. There is substantial debate whether one should adjust for multiple comparisons in our study. As an intervention like a physical activity program is intended to have simultaneous effects on different health outcomes (like body composition, physical activity, fitness and quality of life) and most of these outcomes are to some extent related. The four primary endpoints mentioned in the protocol represent parallel research questions and not different facets of a global hypothesis. Thus, we have not considered one isolated highly significant intervention effect as proof of success of our intervention, but originally expected a promising public health intervention to have beneficial and relevant effects in most, if not all dimensions. All differences in the main outcome measures were in favour of the intervention group, but only the observed effect on aerobic fitness reached statistical significance. Thus, despite the strong result for aerobic fitnessour expectations were not fully met, possibly due also to a lack of statistical power.

## Conclusions

After initial beneficial effects in aerobic fitness, physical activity, body fat and a composite cardiovascular risk score were reached by a multi-component physical activity intervention in school over an academic year, sustained benefits after three years were clearly seen only for aerobic fitness. Although this is highly relevant from a Public Health perspective, longer term interventions throughout the school years are needed to attain persistent beneficial health effects in different relevant dimensions other than fitness.

## Supporting Information

Protocol S1 **Trial protocol.**
(PDF)

Checklist S1 **CONSORT Checklist.**
(DOC)

## References

[1] Beaglehole R, Bonita R, Horton R, Adams C, Alleyne G, et al (2011) Priority actions for the non-communicable disease crisis. Lancet 377: 1438. 21474174 10.1016/S0140-6736(11)60393-0

[2] Andersen LB, Riddoch C, Kriemler S, Hills A (2011) Physical activity and cardiovascular risk factors in children. Br J Sports Med 45: 871–876. 21791456 10.1136/bjsports-2011-090333

[3] Andersen LB, Harro M, Sardinha LB, Froberg K, Ekelund U, et al (2006) Physical activity and clustered cardiovascular risk in children: a cross-sectional study (The European Youth Heart Study). Lancet 368: 299–304. 16860699 10.1016/S0140-6736(06)69075-2

[4] Brage S, Wedderkopp N, Ekelund U, Franks PW, Wareham NJ, et al (2004) Features of the metabolic syndrome are associated with objectively measured physical activity and fitness in Danish children: the European Youth Heart Study (EYHS). Diabetes Care 27: 2141–2148. 15333475 10.2337/diacare.27.9.2141

[5] Lee DC, Sui X, Church TS, Lavie CJ, Jackson AS, et al (2012) Changes in fitness and fatness on the development of cardiovascular disease risk factors hypertension, metabolic syndrome, and hypercholesterolemia. J Am Coll Cardiol 59: 665–672. 22322083 10.1016/j.jacc.2011.11.013 PMC3293498

[6] Tomkinson GR, Olds TS (2007) Secular changes in pediatric aerobic fitness test performance: the global picture. Med Sport Sci 50: 46–66. 17387251 10.1159/000101075

[7] Sisson SB, Katzmarzyk PT (2008) International prevalence of physical activity in youth and adults. Obes Rev 9: 606–614. 18647243 10.1111/j.1467-789X.2008.00506.x

[8] Ekelund U, Luan J, Sherar LB, Esliger DW, Griew P, et al (2012) Moderate to vigorous physical activity and sedentary time and cardiometabolic risk factors in children and adolescents. JAMA 307: 704–712. 22337681 10.1001/jama.2012.156 PMC3793121

[9] van Sluijs EM, McMinn A (2010) Preventing obesity in primary schoolchildren. Br Med J 340: c819. 20179127 10.1136/bmj.c819

[10] Oude Luttikhuis H, Baur L, Jansen H, Shrewsbury VA, O'Malley C, et al. (2009) Interventions for treating obesity in children. Cochrane Database Syst Rev: CD001872. 10.1002/14651858.CD001872.pub2 19160202

[11] Singh AS, Mulder C, Twisk JW, van Mechelen W, Chinapaw MJ (2008) Tracking of childhood overweight into adulthood: a systematic review of the literature. Obes Rev 9: 474–488. 18331423 10.1111/j.1467-789X.2008.00475.x

[12] Kriemler S, Meyer U, Martin E, van Sluijs EM, Andersen LB, et al (2011) Effect of school-based interventions on physical activity and fitness in children and adolescents: a review of reviews and systematic update. Br J Sports Med 45: 923–930. 21836176 10.1136/bjsports-2011-090186 PMC3841814

[13] Dobbins M, De Corby K, Robeson P, Husson H, Tirilis D (2009) School-based physical activity programs for promoting physical activity and fitness in children and adolescents aged 6–18. Cochrane Database Syst Rev: Cd007651. 10.1002/14651858.CD007651 19160341

[14] Kriemler S, Zahner L, Schindler C, Meyer U, Hartmann T, et al (2010) Effect of school based physical activity programme (KISS) on fitness and adiposity in primary schoolchildren: cluster randomised controlled trial. Br Med J 340: c785–c785. 20179126 10.1136/bmj.c785 PMC2827713

[15] Zahner L, Puder JJ, Roth R, Schmid M, Guldimann R, et al (2006) A school-based physical activity program to improve health and fitness in children aged 6–13 years (“Kinder-Sportstudie KISS”): study design of a randomized controlled trial [ISRCTN15360785]. BMC Public Health 6: 147. 16756652 10.1186/1471-2458-6-147 PMC1513202

[16] de Onis M, Onyango AW, Borghi E, Siyam A, Nishida C, et al (2007) Development of a WHO growth reference for school-aged children and adolescents. Bull World Health Organ 85: 660–667. 18026621 10.2471/BLT.07.043497 PMC2636412

[17] Lohman TG, Roche AF, Martorell R (1988) Anthropometric Standardization Reference Material. Champaign: Human Kinetics.

[18] Leger LA, Mercier D, Gadoury C, Lambert J (1988) The multistage 20 metre shuttle run test for aerobic fitness. J Sports Sci 6: 93–101. 3184250 10.1080/02640418808729800

[19] Freedson P, Pober D, Janz KF (2005) Calibration of accelerometer output for children. Med Sci Sports Exerc 37: S523–S530. 16294115 10.1249/01.mss.0000185658.28284.ba

[20] Landgraf JM, Maunsell E, Speechley KN, Bullinger M, Campbell S, et al (1998) Canadian-French, German and UK versions of the Child Health Questionnaire: methodology and preliminary item scaling results. Qual Life Res 7: 433–445. 9691723 10.1023/a:1008810004694

[21] National High Blood Pressure Education Program Working Group on High Blood Pressure in Children and Adolescents (2004) The fourth report on the diagnosis, evaluation, and treatment of high blood pressure in children and adolescents. Pediatrics 114: 555–576. 15286277

[22] Eisenmann JC (2008) On the use of a continuous metabolic syndrome score in pediatric research. Cardiovasc Diabetol 7: 17. 18534019 10.1186/1475-2840-7-17 PMC2430947

[23] Woringer V, Schutz Y (2003) [Obesity in Switzerland: body mass index (BMI) percentiles of a child and adolescent population born in 1980 in Lausanne and comparison with Swiss norms (1955)]. Soz Praventivmed 48: 121–132. 12841084 10.1007/s00038-003-0103-5

[24] Tomkinson GR, Leger LA, Olds TS, Cazorla G (2003) Secular trends in the performance of children and adolescents (1980–2000): an analysis of 55 studies of the 20 m shuttle run test in 11 countries. Sports Med 33: 285–300. 12688827 10.2165/00007256-200333040-00003

[25] Kodama S, Saito K, Tanaka S, Maki M, Yachi Y, et al (2009) Cardiorespiratory fitness as a quantitative predictor of all-cause mortality and cardiovascular events in healthy men and women: a meta-analysis. JAMA 301: 2024–2035. 19454641 10.1001/jama.2009.681

[26] Bugge A, El-Naaman B, Dencker M, Froberg K, Holme IM, et al. (2012) Effects of a 3-year intervention: The Copenhagen School Child Intervention Study. Med Sci Sports Exerc [Epub ahead of print]. 10.1249/MSS.0b013e31824bd579 22297806

[27] Kelder SH, Mitchell PD, McKenzie TL, Derby C, Strikmiller PK, et al (2003) Long-term implementation of the CATCH physical education program. Health Educ Behav 30: 463–475. 12929897 10.1177/1090198103253538

[28] Klepp KI, Tell GS, Vellar OD (1993) Ten-year follow-up of the Oslo Youth Study Smoking Prevention Program. Prev Med 22: 453–462. 8415496 10.1006/pmed.1993.1037

[29] Nader PR, Stone EJ, Lytle LA, Perry CL, Osganian SK, et al (1999) Three-year maintenance of improved diet and physical activity: the CATCH cohort. Child and Adolescent Trial for Cardiovascular Health. Arch Pediatr Adolesc Med 153: 695–704. 10401802 10.1001/archpedi.153.7.695

[30] Trudeau F, Laurencelle L, Tremblay J, Rajic M, Shephard RJ (1999) Daily primary school physical education: effects on physical activity during adult life. Med Sci Sports Exerc 31: 111–117. 9927018 10.1097/00005768-199901000-00018

[31] Manios Y, Kafatos A (2006) Health and nutrition education in primary schools in Crete: 10 years follow-up of serum lipids, physical activity and macronutrient intake. Br J Nutr 95: 568–575. 16578934 10.1079/bjn20051666

[32] Trudeau F, Espindola R, Laurencelle L, Dulac F, Rajic M, et al (2000) Follow-up of participants in the Trois-Rivieres Growth and Development Study: Examining their health-related fitness and risk factors as adults. Am J Hum Biol 12: 207–213. 11534017 10.1002/(SICI)1520-6300(200003/04)12:2<207::AID-AJHB6>3.0.CO;2-8

[33] Kafatos I, Manios Y, Moschandreas J, Kafatos A (2007) Health and nutrition education program in primary schools of Crete: changes in blood pressure over 10 years. Eur J Clin Nutr 61: 837–845. 17213871 10.1038/sj.ejcn.1602584

[34] Trudeau F, Laurencelle L, Tremblay J, Rajic M, Shephard RJ (1998) A long-term follow-up of participants in the Trois-Riviäres semi-longitudinal study of growth and development. Pediatr Exerc Sci 10: 366-377.

[35] James J, Thomas P, Kerr D (2007) Preventing childhood obesity: two year follow-up results from the Christchurch obesity prevention programme in schools (CHOPPS). Br Med J (Clin Res Ed) 335: 762. 10.1136/bmj.39342.571806.55 PMC2018766 17923721

[36] Glasgow RE, Vogt TM, Boles SM (1999) Evaluating the public health impact of health promotion interventions: the RE-AIM framework. Am J Public Health 89: 1322–1327. 10474547 10.2105/ajph.89.9.1322 PMC1508772

[37] De Meij JS, Chinapaw MJ, Kremers SP, Van der Wal MF, Jurg ME, et al (2010) Promoting physical activity in children: The stepwise development of the primary school-based JUMP-in intervention applying the RE-AIM evaluation framework. Br J Sports Med 44: 879–887. 19019902 10.1136/bjsm.2008.053827

[38] Collard DC, Chinapaw MJ, Verhagen EA, van Mechelen W (2010) Process evaluation of a school based physical activity related injury prevention programme using the RE-AIM framework. BMC Pediatr 10: 86. 21092316 10.1186/1471-2431-10-86 PMC3004886

[39] Naylor PJ, Macdonald HM, Warburton DE, Reed KE, McKay HA (2008) An active school model to promote physical activity in elementary schools: action schools! BC. Br J Sports Med 42: 338–343. 18272538 10.1136/bjsm.2007.042036

